# The Association Between Shift Work and Migraine Attacks Among Healthcare Workers in the Kingdom of Saudi Arabia

**DOI:** 10.7759/cureus.53315

**Published:** 2024-01-31

**Authors:** Leen S Al Maqwashi, Albaraa M Sufyani, Mawahib M Bichara, Yousef T Rajikhan, Maram Albishri, Nouf A Hamood, Raghad H Al Dligan, Ibrahim Tawhari

**Affiliations:** 1 College of Medicine, Sulaiman Al Rajhi University, Al Bukayriyah, SAU; 2 Faculty of Medicine, King Abdulaziz University, Jeddah, SAU; 3 College of Medicine, Ibn Sina National College for Medical Studies, Jeddah, SAU; 4 College of Medicine, King Khalid University, Abha, SAU; 5 Department of Internal Medicine, King Khalid University, Abha, SAU

**Keywords:** healthcare workers, association study, cross-sectional, kingdom of saudi arabia (ksa), shift work, migraine attacks

## Abstract

Introduction

Migraine, a prevalent condition in Saudi Arabia, is linked to various risk factors, including night shifts. Existing literature, mainly outdated, suggests conflicting findings on the relationship between sleep, night shifts, and migraines. Our study aims to investigate the specific association between shift work and migraine attacks among healthcare workers in the Kingdom of Saudi Arabia (KSA), addressing a notable research gap.

Methodology

This is a cross-sectional study conducted in Saudi Arabia. Data were collected by using a non-probability convenience sampling technique. Data were collected through an online questionnaire and analyzed using SPSS version 26 (IBM Corp., Armonk, NY).

Results

Our study on 342 healthcare workers in the KSA revealed the majority of participants were females (70.5%, n = 241), aged between 25 and 29 years (38.9%, n = 133), with doctors being the predominant profession (51.5%, n = 176). Participants had an average of 5.9 years of healthcare experience. Work shifts included rotating (43.3%, n = 148), day (48%, n = 164), evening (3.8%, n = 13), and night shifts (5%, n = 17). Notably, 89.2% (n = 305) experienced headaches with varying characteristics and triggers. Management strategies included over-the-counter painkillers (56.1%, n = 192) and rest (50.5%, n = 173). Gender was significantly associated with migraines (p = 0.020), while night shift frequency and years in health care showed no significant associations. Higher weekly working hours relate significantly to migraines (p = 0.034).

Conclusion

Our study highlights a significant association between migraines and gender, with females being more prone. Night shift frequency and years in health care showed no significant associations, while higher weekly working hours were linked to migraines.

## Introduction

Background

Migraine is a complex disease characterized by a throbbing headache, usually localized on one side of the head, of moderate to severe intensity lasting four to 72 hours and accompanied by photophobia and/or gastrointestinal symptoms (for example, nausea and vomiting). In about 15% to 1/3 of migraine cases, the headache is preceded or accompanied by an aura, which is a collection of focal, neurological, visual, sensory, speech, and/or motor symptoms. It occurs gradually, spreads, and then disappears. And often precedes the headache phase [1]. Migraines are common in Saudi Arabia. The prevalence of migraine in different parts of the kingdom ranges from 25% to 78.5% [2]. There are many risk factors that contribute to migraines; one of them is the night shift. Many essential professions are required to work continuously and provide quality service to their clients around the clock, but this is limited by personal, financial, and organizational factors [3,4]. It is estimated that more than 20 million Americans and Europeans work night shifts, and healthcare workers make up a large part of this [5]. This is partly related to circadian rhythm disturbances and sleep-related disorders [6,7]. Unfortunately, there is a lack of studies about our topic in the Kingdom of Saudi Arabia (KSA). There is only one study that was conducted in 2021 that has limitations in methodology, such as a low number of participants (<300) and a single region [8]. Also, there are too few studies that talk specifically about the relationship between migraines and shift work. Although there is a clear relationship between sleep and headaches [9-12], other studies suffer from limitations in methodology, such as a low number of participants (<300) [12-14]. Also, most of the studies are outdated from 2004 to 2019 [9-11,13-20]. A study from Norway reported that work schedule and number of night shifts do not affect the occurrence of migraine but rather sleep disorder (insomnia disorder) [15]. On the other hand, there is a study in China that reported that an increase in night shifts could possibly cause an association with the prevalence of headaches [20]. For that, our aim is to see the association between shift work and migraine attacks among healthcare workers in the KSA.

The main objective of the study is to determine the association between shift work and migraine attacks among healthcare workers in the KSA. The secondary objectives are to determine the prevalence of migraine attacks among healthcare workers on night and day shifts in the KSA, to assess the impact of migraine attacks on the productivity and general well-being of healthcare workers on shift work, and to provide insights and recommendations to healthcare workers in healthcare institutions for treatment and prevention of migraine attacks in healthcare workers who work shifts.

## Materials and methods

Study settings

The study was conducted in cities in Saudi Arabia after obtaining approval from the Research Ethics Committee at King Khalid University (HAPO-06-B-001). The study was conducted between November and December 2023. All healthcare workers living in Saudi Arabia who agreed to participate in the study were included in the study. The non-healthcare workers and those who refused to participate were excluded from the study.

Study design

This was an observational cross-sectional study in which adult healthcare workers in Saudi Arabia were invited to participate in answering a self-administered questionnaire.

Recruitment of participants

As part of the study, healthcare workers in the KSA were approached and asked to participate. Each individual research participant was informed that completing and returning the questionnaire constituted written consent and agreement to participate. Each participant was assured that the survey was anonymous, that they would not be asked for their name at any time, and that the information they provided would only be used for this research. Finally, they were told that participation was completely optional.

Sampling method

A non-probability-based convenience sampling technique was used in the study. A self-administered questionnaire was used to collect the data (a data collection tool where respondents were asked to provide answers to a series of questions). Using this sampling strategy, participants were selected based on their willingness to participate in the study and their ability to meet the study's inclusion criteria. With this strategy, the researcher managed to attract a significant part of the target group.

Sample size calculation

The Epi Info program (CDC, Atlanta, GA) was used for sample size calculation. The minimum recommended size (n) for this study was 300 based on the following assumption: E = margin of error of 5%, confidence level of 95%, and the number of healthcare workers living in Saudi Arabia.

Data collection tools

The study was conducted through an online self-administered questionnaire prepared in English after reading and accepting the informed consent and distributed via an anonymous online survey instrument, which targeted Saudi and non-Saudi healthcare workers living in the KSA. A pilot study was conducted and applied by the investigators to collect demographic data (not including the name), the number of night shifts per month, screening tools for migraine, and the risk factors of headaches. The answers were evaluated in a survey using yes/no, multiple choice questions, open answers, and selected questions.

Data entry and statistical analysis

Statistical data entry and analysis were performed using SPSS version 26 (IBM Corp., Armonk, NY). The analysis involved both descriptive statistics and inferential statistics according to the required purpose of each relationship. Frequency distributions were obtained, and descriptive statistics were calculated. Another level of data analysis, i.e., the chi-square test, was used to test some associations.

## Results

Table 1 shows the characteristics of the 342 participants. The majority of participants were females (70.5%), compared to 29.5% of males. In terms of age, the largest proportion falls within the 25-29 years category (38.9%), followed by the 30-39 years group (28.7%). Regarding professional roles, doctors constitute the majority at 51.5%, followed by nurses at 18.4%. The distribution of participants based on years of experience in health care revealed a mean of 5.9 years, ranging from less than one year to 40 years. The participants' work shifts vary, with 43.3% engaged in rotating shifts, 48% in day shifts (7 am to 3 pm), 3.8% in evening shifts (3 pm to 11 pm), and 5% in night shifts (11 pm to 7 am). Among those working night shifts, the frequency was diverse, ranging from never (25.4%) to always (4.4%). The average number of hours worked per week was 37.9, with a standard deviation of 16.7 hours and a broad range from 0 to 104 hours.

**Table 1:** Sociodemographic and working shift-related parameters of participants

		Frequency (n = 342)	Percent
Gender	Female	241	70.5
	Male	101	29.5
Age	18-24 years	67	19.6
	25-29 years	133	38.9
	30-39 years	98	28.7
	40-49 years	36	10.5
	>50 years	8	2.3
Specialty	Doctor	176	51.5
	Nurse	63	18.4
	Other	36	10.5
	Pharmacist	24	7
	Lab technician	18	5.3
	Anesthesiologist	13	3.8
	Paramedic	12	3.5
No. of years of experience in health care	Mean (SD)	5.9 (6.3) years	
	Range	<1-40 years	
Type of shifts	Rotating shifts	148	43.3
	Dayshift (7 am to 3 pm)	164	48.0
	Evening shift (3 pm to 11 pm)	13	3.8
	Night shift (11 pm to 7 am)	17	5.0
How frequently do you work in the night shift?	Never	87	25.4
	Rarely (1-2 times per month)	76	22.2
	Sometimes (1-2 times per week)	107	31.3
	Often (3-4 times per week)	57	16.7
	Always	15	4.4
Average hour of work/week	Mean (SD)	37.9 (16.7) hours	
	Range	0-104 hours	

Table 2 shows the features and characteristics of migraine pain among the participants. The majority (89.2%) have experienced headaches, with only 10.8% reporting no history of headaches. Concerning professional diagnosis, 67.3% have not been diagnosed by a healthcare professional, while 21.9% have received a formal diagnosis for migraines. The severity of migraines, as self-assessed on a 1-10 scale, had a mean of 5.1, with a standard deviation of 2.4, indicating a moderate level of intensity. Participants reported feeling headache pain in various areas, with 31.3% experiencing pain in the back of the head/forehead, 19.3% around one eye, 22.5% on one side of the head, and 16.1% behind the forehead/cheekbones. Regarding aura before a headache, 29.2% reported experiencing it sometimes, while 10.8% reported always having aura symptoms. The frequency of headache symptoms varied, with 34.2% experiencing them less than once a month, 33.3% experiencing them one to three times a month, and 13.5% experiencing them weekly. Most respondents (53.8%) reported headaches lasting less than four hours, while 30.7% reported headaches lasting four to 72 hours. A significant portion of participants (63.7%) reported that their headache attacks increased with schedule changes, highlighting a potential correlation between shift work and migraines. Additionally, 43.9% reported reduced productivity during the last three months due to migraines, with varying durations. Finally, 44.7% of participants reported having family members who also experience migraine attacks.

**Table 2:** Features and characteristics of migraine pain in our sample

		Frequency (n = 342)	Percent
Ever experienced a headache	No	37	10.8
	Yes	305	89.2
Migraine diagnosed by healthcare professional	No	230	67.3
	Yes	75	21.9
Severity of migraine on a 1-10 scale	Mean (SD)	5.1 (2.4)	
	Range	1-10	
Where do you feel headache pain most often?	Back of head/forehead	107	31.3
	Around one eye	66	19.3
	One side of the head	77	22.5
	Behind the forehead/cheekbones	55	16.1
Experienced aura before a headache?	Never	182	5.0
	Sometimes	100	29.2
	Often	17	89.2
	Always	6	10.8
How often do you experience these symptoms?	Daily	1	.3
	Weekly	46	13.5
	Several times a week	27	7.9
	Less than once a month	117	34.2
	1-3 times a month	114	33.3
How long does a headache usually last?	<4 hours	184	53.8
	4-72 hours	105	30.7
	> 3 days	16	4.7
Headache attacks increase by schedule changes?	No	87	25.4
	Yes	218	63.7
Experienced reduced productivity during last 3 months	None	87	25.4
	1-2 days	150	43.9
	3-5 days	46	13.5
	>6 days	22	6.4
Do any family members (parents, siblings) experience migraine attacks?	No	152	44.4
	Yes	153	44.7

**Figure 1:**
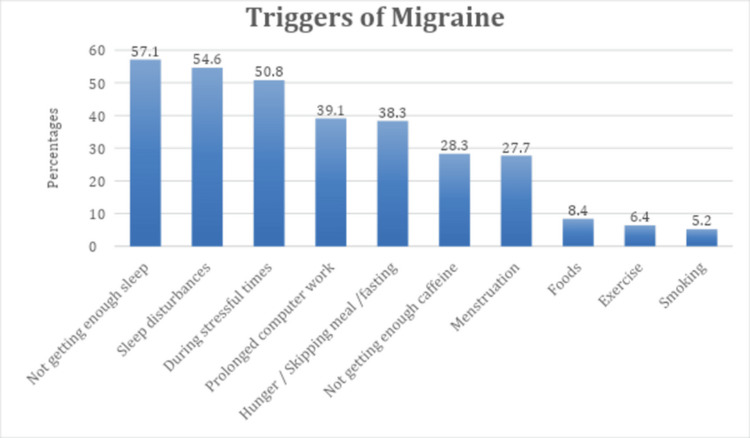
Different triggers of migraine pain in our sample

Figure 1 shows the reported triggers of migraine pain among healthcare workers. The most common triggers include inadequate sleep (195, 57.1%), followed by sleep disturbances (187, 54.6%), and stress (174, 50.8%). Prolonged computer work (134, 39.1%) and hunger (131, 38.3%) are also notable contributors. Less frequently mentioned triggers include caffeine, menstruation, foods, exercise, and smoking.

**Figure 2:**
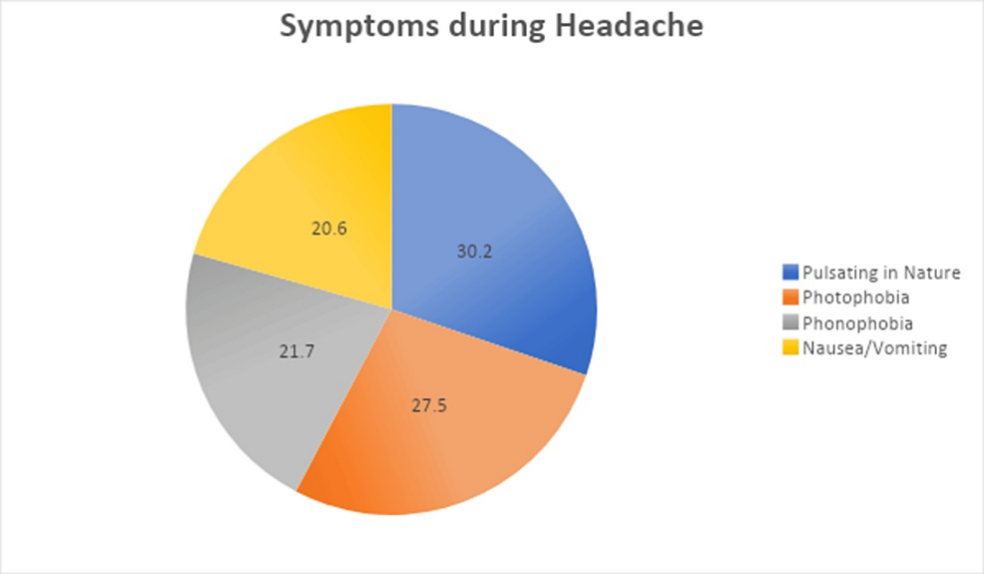
Different symptoms during a migraine attack in our sample

Figure 2 shows the symptoms experienced during migraine attacks among healthcare workers. The most prevalent symptom was a pulsating nature of pain in 166 (30.2%) participants, followed by sensitivity to light (photophobia) in 151 (27.5%) participants, and sensitivity to sound (phonophobia) in 119 (21.7%) participants. Nausea and vomiting were also reported, albeit at a slightly lower frequency (113, 20.6%).

**Figure 3:**
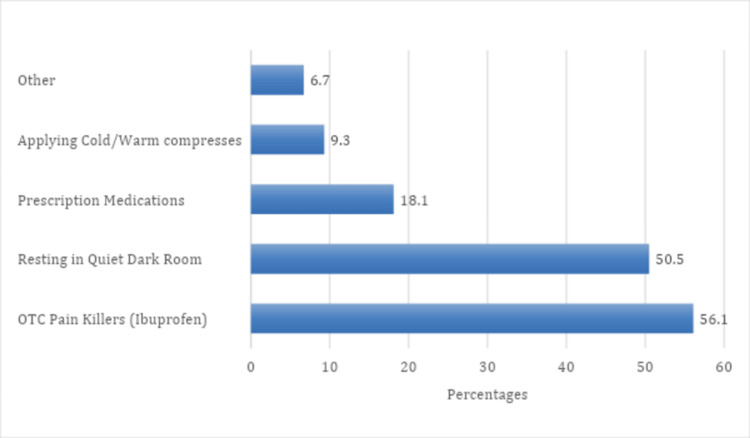
Management of acute attack of migraine OTC: over-the-counter.

Figure 3 shows the strategies employed by participants to manage acute migraine attacks. The most common approach is the use of over-the-counter painkillers, with ibuprofen being mentioned by 192 (56.1%) participants. Resting in a quiet dark room is also a prevalent method, reported by 173 (50.5%) participants. Prescription medications are utilized by 62 (18.1%), and applying cold/warm compresses is a strategy for 32 (9.3%) participants. A smaller percentage (23, 6.7%) indicated using other methods for managing acute migraine attacks.

Table 3 shows the association of sociodemographic and work shift-related factors with migraine pain among 342 healthcare workers. Notably, gender shows a significant association (p = 0.020), with 91.7% of females and 83.2% of males reporting migraines. No significant associations were found for age groups or healthcare specialties. However, the type of shift yielded no significant association (p = 0.275). Interestingly, the frequency of night shifts (p = 0.799) and the number of years in health care (p = 0.824) did not show significant associations. Notably, the mean number of hours worked per week demonstrated a significant association (p = 0.034), indicating higher hours among those with migraines (38.6 vs. 32.3).

**Table 3:** Association of sociodemographic features and work shift-related factors with migraine pain Fisher’s exact test and independent t-test were used.

			Suffered with migraine		Sig. value
			No	Yes	
Gender	Female	N	20	221	0.020
		%	8.3%	91.7%	
	Male	N	17	84	
		%	16.8%	83.2%	
Age	18-24 years	N	10	57	0.271
		%	14.9%	85.1%	
	25-29 years	N	10	123	
		%	7.5%	92.5%	
	30-39 years	N	11	87	
		%	11.2%	88.8%	
	40-49 years	N	4	32	
		%	11.1%	88.9%	
	>50 years	N	2	6	
		%	25.0%	75.0%	
Specialty in health care	Anesthesiologist	N	2	11	0.570
		%	15.4%	84.6%	
	Doctor	N	18	158	
		%	10.2%	89.8%	
	Lab technician	N	1	17	
		%	5.6%	94.4%	
	Nurse/paramedics	N	12	63	
		%	16.0%	84.0%	
	Pharmacist	N	2	22	
		%	8.3%	91.7%	
	Other	N	2	34	
		%	5.6%	94.4%	
Type of shift	Dayshift (7 am to 3 pm)	N	23	141	0.275
		%	14.0%	86.0%	
	Evening shift (3 pm to 11 pm)	N	1	12	
		%	7.7%	92.3%	
	Night shift (11 pm to 7 pm)	N	2	15	
		%	11.8%	88.2%	
	Rotating shifts	N	11	137	
		%	7.4%	92.6%	
Frequency of night shifts	Always	N	1	14	0.799
		%	6.7%	93.3%	
	Never	N	12	75	
		%	13.8%	86.2%	
	Often (3-4 times per week)	N	7	50	
		%	12.3%	87.7%	
	Rarely (1-2 times per month)	N	8	68	
		%	10.5%	89.5%	
	Sometimes (1-2 times per week)	N	9	98	
		%	8.4%	91.6%	
No. of years in health care		Mean	6.1	5.9	0.824
		SD	7.7	6.1	
No. of hours of work/week		Mean	32.3	38.6	0.034
		SD	18.7	16.3	

## Discussion

Migraine, a prevalent and complex condition, often accompanied by aura, poses a significant health concern in Saudi Arabia, with the prevalence ranging from 25% to 78.5%. Albalawi et al. have shown that Saudi Arabia had approximately a 77.2% prevalence of all types of headaches, with a 25% prevalence of migraines [2,21]. Night shift work, common among healthcare professionals, is a potential risk factor for migraines, linked to circadian rhythm disturbances. Leso et al. (2020) have shown that the increased frequency of night shifts, particularly more than eight per month, significantly raises the risk of migraine in nurses. Those working over eight night shifts show a 29.4% risk compared to 18.9% for those with fewer night shifts [22]. Despite limited local studies, existing research has shown varying results. Our study addresses this gap by examining the association between shift work and migraine attacks among healthcare workers in Saudi Arabia. Our findings provide valuable insights into the prevalence, characteristics, triggers, symptoms, and management strategies of migraines in this specific occupational group.

The demographic and professional profile of the participants is noteworthy. The predominance of female participants (70.5%) aligns with the gender distribution in the healthcare sector and the more prevalent gender affected with migraine. Similarly, Rossi et al. (2022) have shown that the global prevalence of migraine is higher (20.7%) in women as compared to 9.7% in men [23]. The age distribution reveals a concentration in the 25-29 years category (38.9%), possibly reflecting the age distribution of healthcare professionals in the KSA and the prevalence of migraine in that age group. Bigal et al. (2006) have shown that migraine prevalence was highest between ages 30 and 39 years while Victor et al. (2010) have shown a bimodal distribution in both sexes (peaking in the late teens and 20s and around 50 years of age) [24,25]. The majority being doctors (51.5%) underscores the diverse roles within the healthcare workforce studied. The average of 5.9 years of experience demonstrates a relatively early to mid-career stage for most participants. Variability in work shifts, including rotating shifts and night shifts, is consistent with the dynamic nature of healthcare operations.

When we dive into the characteristics of migraine pain, there is a significant prevalence of migraine among healthcare workers. Notably, 89.2% of participants reported experiencing headaches, with a mean severity of 5.1 on a 1-10 scale. A study by Choudry et al. (2022) shows that the total prevalence of migraine in physicians and medical students is 24.4% [26]. The variety of reported headache locations and accompanying symptoms such as aura, nausea, and vomiting align with classical migraine features documented in the medical literature. The duration of headaches and their association with schedule changes, reported by 63.7% of participants, suggests a potential link between migraines and shift work. Similarly, previous studies show that there is an association between shift work and migraine attacks [15,16].

Various identified triggers resonate with existing literature on migraine triggers. Sleep-related factors, such as inadequate sleep and sleep disturbances, were major contributors, consistent with studies highlighting the impact of sleep disruption on migraine susceptibility. Lin et al. (2016) have shown that sleep problems like disturbed or inadequate sleep are a particularly common problem among migraineurs (children or adults), affecting 30% to 50% of migraine patients [27]. Stress and prolonged computer work also emerged as common triggers, emphasizing the multifactorial nature of migraines in healthcare settings. Stubberud et al. (2021) have shown that higher migraine frequency is associated with higher levels of perceived stress [28]. The reported symptoms during migraine attacks align with classical manifestations, with pulsating pain, sensitivity to light and sound, and nausea/vomiting being prevalent.

Various strategies are employed by participants to manage acute migraine attacks. Over-the-counter painkillers, particularly ibuprofen, and rest in a quiet dark room are the primary interventions. A study by Demaagd et al. (2008) shows that nonsteroidal anti-inflammatory drugs are used in the abortive management of migraine [29]. The prevalence of self-medication aligns with the literature, emphasizing the need for healthcare professionals to seek effective management strategies for migraines to ensure optimal productivity and well-being.

Regarding the association between sociodemographic and work shift factors with migraine pain, the significant association between gender and migraines (p = 0.020) is consistent with previous research highlighting a higher prevalence of migraines in females [30]. The lack of significant associations with age and specialty is intriguing and warrants further exploration. In contrast, a previous study by Xie et al. (2020) shows that both types of primary headache were more prevalent in nurses than in doctors (migraine: 29.2% vs. 21.7%, p = 0.045; tension-type headache: 24.7% vs. 23.3%, p = 0.715) [31]. Interestingly, the type of shift did not exhibit a significant association, challenging existing literature and suggesting a correlation between shift work and migraines. A previous study by Sandoe et al. (2019) shows that shift work appeared to be associated with chronification of migraine [16]. However, the significant association between the number of hours worked per week and migraines (p = 0.034) suggests that increased working hours may contribute to migraine susceptibility, aligning with studies linking long work hours to adverse health outcomes.

Limitations and future directions

Limitations include the cross-sectional design, which precludes establishing causation, and reliance on self-reported data, potentially introducing recall bias. Future research should explore longitudinal associations, considering additional factors such as sleep quality, stress levels, and genetic predispositions.

## Conclusions

Our study sheds light on the complex interplay between shift work and migraines among healthcare workers in the KSA. The significant prevalence of migraines, diverse triggers, and association with working hours underscore the importance of tailored interventions to mitigate the impact on healthcare professionals' well-being and productivity. The findings contribute to the evolving understanding of migraines in the unique context of healthcare work in the KSA, urging further research for comprehensive insights and targeted interventions.

## Appendices

**Table 4:** Questionnaire

Questionnaire	
Section A	
1. What is your age?	• (18 - 24) • (25 - 29) • (30 - 39) • (40 - 49) • (More than 50)
2. Gender?	• Female • Male
3. What is your specialty?	..............................................
4. How many years have you been working in health care?	.............................................
5. What is your primary work shift?	• Dayshift (7 am - 3 pm) • Evening shift (3 pm - 11 pm) • Night shift (11 pm - 7 am) • Rotating shifts • Others:..........
6. How frequently do you work night shifts?	• Never • Rarely (1-2 times per month) • Sometimes (1-2 times per week) • Often (3-4 times per week) • Always
7. On average, how many hours do you work per week?	.............................................
8. Have you ever had a headache?	• Yes • No (End of survey)
Section B	
1. Have you ever been diagnosed with migraine by a healthcare professional?	• Yes • No
2. Where do you feel headache pain most often?	• Usually behind the forehead and/or cheekbones • Around one eye • One side of the head • Around the back of the head and the forehead

3. Do any of the following bring on/ trigger your headaches? (Select all triggers)	⬜ Foods ⬜ Not getting enough caffeine ⬜ Hunger/skipping meal/fasting ⬜ Smoking ⬜ During stressful times ⬜ Menstruation ⬜ Exercise ⬜ Prolonged computer work ⬜ Sleep disturbances ⬜ Not getting enough sleep
4. On a scale of 1 to 10, how severe are your migraine attacks, with 1 being mild and 10 being severe?	1 2 3 4 5 6 7 8 9 10
5. Do you experience any of these symptoms during your headaches?	• Pulsating in nature • Sensitivity to light (photophobia) • Sensitivity to sound (phonophobia) • Nausea or vomiting
6. Aura (visual disturbances, sensory changes, or speech difficulties) preceding the headache?	•Never • Sometimes • Often • Always
7. How often do you experience these symptoms?	• Less than once a month • 1-3 times a month • Weekly • Several times a week • Daily
8. How long does a headache usually last?	• Less than 4 hours • 4-72 hours • More than 3 days
9. Do your headache attacks increase by schedule changes?	• Yes • No

10. In the last 3 months, how many days did you experience reduced productivity at work because of your headaches?	• None • 1-2 days • 3-5 days • More than 6 days
11. How do you typically manage your migraine attacks? (Select all that apply)	⬜ Over-the-counter pain relievers (e.g., ibuprofen) ⬜ Prescription medications ⬜ Resting in a quiet, dark room ⬜ Applying cold or warm compresses ⬜ Other
12. Do any of your close family members (parents, siblings) experience migraine attacks?	• Yes • No
